# Impact of type 2 diabetes mellitus on in-hospital outcomes in patients with ST-elevation myocardial infarction: A nationwide study in Germany

**DOI:** 10.1016/j.metop.2026.100462

**Published:** 2026-03-25

**Authors:** Volker H. Schmitt, Omar Hahad, Visvakanth Sivanathan, Christoph Brochhausen, Christine Espinola-Klein, Thomas Münzel, Philipp Lurz, Tommaso Gori, Lukas Hobohm, Karsten Keller

**Affiliations:** a Department of Cardiology, University Medical Center of the Johannes Gutenberg-University of Mainz, Germany; b German Center for Cardiovascular Research (DZHK), Partner Site Rhine Main, Mainz, Germany; c Department of Gastroenterology, University Medical Center of the Johannes Gutenberg-University of Mainz, Germany; d Institute of Pathology, Medical Faculty Mannheim, Heidelberg University, Germany; e Center for Thrombosis and Hemostasis (CTH), University Medical Center of the Johannes Gutenberg-University of Mainz, Germany

**Keywords:** ST-Elevation myocardial infarction, Type 2 diabetes mellitus, Clinical outcome, Mortality, Epidemiology

## Abstract

**Background:**

Type 2 diabetes mellitus (T2DM) represents a substantial health burden especially regarding cardiovascular diseases. In this context, the presence of T2DM adversely affects both disease emergence and progression. Therefore, this study investigated the impact of T2DM on outcomes in patients hospitalized for ST-elevation myocardial infarction (STEMI).

**Methods:**

We used the nationwide German inpatient statistics of the years 2005-2022 for statistical analysis. Hospitalizations of patients who were admitted due to STEMI in German hospitals were included in this study. The impact of T2DM on in-hospital adverse events was evaluated, whereas patients with type 1 diabetes mellitus (T1DM) were excluded.

**Results:**

Overall, data on 1,590,775 hospitalizations of patients admitted due to STEMI were included; among these 379,346 (23.8%) patient-cases had T2DM. STEMI patients with T2DM were older (71.0 [62.0-79.0] vs. 66.0 [55.0-77.0] years, P < 0.001) and had an aggravated comorbidity burden. STEMI patients with T2DM were less often treated with percutaneous coronary intervention (61.2% vs. 66.7%, P < 0.001), whereas coronary-artery bypass graft was more often performed (5.0% vs. 3.9%, P < 0.001). The in-hospital case-fatality was significantly higher in T2DM patients (16.0% vs. 13.3%, P < 0.001).

In multivariable analysis, T2DM was independently associated with lower usage of percutaneous intervention (OR 0.89 [95%CI 0.89-0.90], P < 0.001) and increased case-fatality (OR 1.03 [95%CI 1.01-1.04], P < 0.001).

**Conclusion:**

In STEMI patients, T2DM was associated with an increased case-fatality and higher complication rate in the light of an underuse of interventional revascularization.

## 1 Introduction

Type 2 diabetes mellitus (T2DM) represents a major risk factor for the development of end organ damage, cardiovascular diseases and mortality [[1], [2], [3], [4]]. By influencing the homeostasis of several biosystems like the inflammatory and the immune system [4,5], T2DM impacts on morbidity and mortality and it has the same relevance for prognosis as prevalent disease [[6], [7], [8], [9]]. Worldwide, T2DM has become epidemic with rising incidence [10]. T2DM is a main risk factor in the development of coronary artery disease and has a vast impact on clinical outcome of patients suffering from acute coronary syndrome [2,[11], [12], [13], [14], [15], [16]], especially in ST-elevation myocardial infarction (STEMI). Several studies reported that the prognosis of STEMI patients was afflicted by T2DM [[17], [18], [19], [20], [21], [22], [23], [24]]. Fortunately, due to improved medical care, a decrease of mortality was achieved in patients with T2DM over the past decades, although the incidence of the disease increased during the same period [25]. However, despite all achievements and progress in treatment approaches, patients with diabetes mellitus are still affected by a considerably increased risk for cardiovascular diseases like coronary artery disease, heart failure, pulmonary embolism, peripheral artery disease and stroke [6,8,26,27]. In this context, diabetes mellitus was shown to cause a doubled risk for coronary artery disease, ischemic stroke and cardiovascular mortality [28]. Already in the early 1990s a worse clinical outcome of patients with T2DM was reported after myocardial infarction [29]. In patients suffering from STEMI, diabetes mellitus leads to worse clinical disease course and outcome [17] as it accelerates the progression of coronary atherosclerosis, favours thrombogenicity and is associated with an increased risk for stent thrombosis and cardiovascular adverse events following percutaneous coronary intervention (PCI) [[30], [31], [32]]. Despite advances in reperfusion and pharmacological therapies, the impact of T2DM on in-hospital outcomes in STEMI remains insufficiently characterized [[33], [34], [35], [36], [37]]. The key objectives of our study were to investigate time-trends in revascularization strategies and differences in revascularization treatments between STEMI patients with and without T2DM, impact of T2DM on outcomes inclusive short-term survival and sex-specific differences in outcomes. Therefore, this nationwide study aimed to investigate the impact of T2DM on clinical outcomes and time trends among all patients hospitalized for STEMI in Germany over an 18-year observation period.

## 2 Materials and methods

In the present study, the German nationwide inpatient statistics was analysed assessing all hospitalizations due to STEMI within the years 2005-2022 (source: Research Data Center of the Federal Statistical Office and the Statistical Offices of the federal states, DRG Statistics 2005-2022, and own calculations). Patients’ diagnoses are coded according to ICD-10-GM (International Classification of Diseases, 10th Revision with German Modification) and diagnostical, surgical and interventional procedures are coded with the OPS codes (surgery, diagnostic and procedures codes [Operationen-und Prozedurenschlüssel]). The Federal Statistical Office of Germany (Statistisches Bundesamt, Wiesbaden, Germany) gathers all treatment data from nearby all inpatient cases of Germany (processed according to the diagnosis related groups [DRG] system) [38,39].

In the present study, all hospitalization-cases of STEMI within the timeframe of the years 2005 to 2022 were included using the diagnostic codes for STEMI (ICD-codes I21.0, I21.1, I21.2 and I21.3). Cases which were additionally coded as type 1 diabetes mellitus (ICD-code E10) were excluded. After stratification of the STEMI patients included based on the presence of T2DM (ICD-code E11), baseline parameters including cardiovascular risk factors and cardiovascular comorbidities, use of reperfusion treatments, age, gender as well as the occurrence of adverse in-hospital events and in-hospital case-fatality rate were analysed. In addition, we investigated time-trends on hospitalization rate, in-hospital case-fatality rate, in-hospital adverse events and revascularization treatments.

As patients’ characteristics, comorbidities, conditions, diagnostical, surgical and interventional procedures were coded from the hospitals, besides a few missings in the gender coding there are no missings in the dataset of the German nationwide inpatient statistics due to the data structure since the variables were coded or not.

### 2.1 Definitions

Obesity was defined as body mass index ≥30 kg/m^2^ according to the recommendations of the WHO (World Health Organization) [40]. Recurrent MI was defined as a recurrent MI event during the first 4 weeks after a first MI event. Shock as well as cardio-pulmonary resuscitation were defined according to current European guidelines [[41], [42], [43]].

### 2.2 Study endpoints and in-hospital adverse events

The primary study outcome was defined as case-fatality comprising death of all-causes during in-hospital stay (in-hospital death). Secondary study outcomes were occurrence of adverse in-hospital events such as recurrent MI (ICD-code I22), pneumonia (ICD-codes J12-J18), deep venous thrombosis and/or thrombophlebitis of the leg veins (ICD-code I80), pulmonary embolism (ICD-code I26), acute kidney injury (ICD-code N17), stroke (ischemic and hemorrhagic stroke, ICD-codes I61-64), intracerebral bleeding events (ICD-code I61), gastro-intestinal bleeding (ICD-codes K920-K922), and transfusion of blood components (OPS code 8-800).

### 2.3 Ethical aspects

Since the investigators had no direct access to data of individual patients, approval by an ethical committee and informed consent were not required in accordance with the German law.

### 2.4 Statistical methods

Descriptive statistics regarding the comparison of relevant patient-characteristics of STEMI patients with and without T2DM were presented as median and interquartile range (IQR) or absolute numbers and corresponding percentages. For continuous variables, the Mann-Whitney-U test was used, whereas categorical variables were assessed with help of Fisher's exact or the chi^2^ test, as appropriate.

Total hospitalization rate for STEMI with T2DM related to all hospitalized STEMI cases (with and without T2DM) and relative mortality rate (case-fatality rate), recurrent MI, the use of interventional reperfusion treatments as well as coronary artery bypass graft (CABG) and rate of adverse in-hospital events were descriptively illustrated in figures.

Univariate and multivariate logistic regression models were analysed to investigate the impact of T2DM on adverse in-hospital events and on in-hospital death in STEMI patients. Results were presented as odds ratio (OR) and 95% confidence interval (CI). The multivariate regression models were adjusted for age, sex, cancer, heart failure, chronic obstructive pulmonary disease, essential arterial hypertension, acute and chronic kidney disease, atrial fibrillation/flutter, and hyperlipidaemia.

The software SPSS® (version 20.0; SPSS Inc., Chicago, Illinois, USA) was used for computerised analysis. P values of <0.05 (two-sided) were considered to be statistically significant.

## 3 Results

During the observational study period including the years 2005-2022, a total of 1,590,775 hospitalization cases of patients with STEMI (excluding patients with type 1 diabetes mellitus) were counted in Germany (32.4% females, 42.8% aged ≥70 years). Of these, 379,346 patient-cases (23.8%) were coded for T2DM. Overall, 221,673 STEMI patients died during hospitalization (accounting for a 13.9% case-fatality rate) (Table 1).

**Table 1:** Baseline characteristics, medical history, presentation and outcome of the included 1,590,775 hospitalization cases of patients admitted due to ST-elevation myocardial infarction (STEMI) stratified according to the presence of type 2 diabetes mellitus (T2DM) (excluding patients with T1DM).

Parameters	STEMI patients without T2DM (n = 1,211,429; 76.2%)	STEMI patients with T2DM (n = 379,346; 23.8%)	P-value
Age	66.0 (55.0-77.0)	71.0 (62.0-79.0)	**<0.001**
Age ≥70 years	480,429 (39.7%)	200,523 (52.9%)	**<0.001**
Female sex	372,493 (30.7%)	143,011 (37.7%)	**<0.001**
In-hospital stay (days)	6 (4-10)	7 (4-13)	**<0.001**
**Traditional cardiovascular risk factors**			
Obesity	89,606 (7.4%)	55,413 (14.6%)	**<0.001**
Essential arterial hypertension	608,659 (50.2%)	231,066 (60.9%)	**<0.001**
Hyperlipidemia	524,783 (43.3%)	170,968 (45.1%)	**<0.001**
**Comorbidities**			
Peripheral artery disease	37,795 (3.1%)	27,974 (7.4%)	**<0.001**
Heart failure	382,447 (31.6%)	166,012 (43.8%)	**<0.001**
Cancer	29,573 (2.4%)	10,568 (2.8%)	**<0.001**
Atrial fibrillation/flutter	167,733 (13.8%)	78,344 (20.7%)	**<0.001**
Chronic obstructive pulmonary disease	60,780 (5.0%)	26,441 (7.0%)	**<0.001**
Renal failure (acute or chronic renal failure)	198,769 (16.4%)	121,815 (32.1%)	**<0.001**
**Treatment**			
Cardiac catheter	878,125 (72.5%)	256,328 (67.6%)	**<0.001**
Percutaneous coronary intervention	808,016 (66.7%)	232,294 (61.2%)	**<0.001**
Coronary-artery bypass graft	46,875 (3.9%)	18,836 (5.0%)	**<0.001**
Intensive care unit	477,438 (39.4%)	157,898 (41.6%)	**<0.001**
**Adverse events during hospitalization**			
In-hospital death	161,148 (13.3%)	60,525 (16.0%)	**<0.001**
Cardiopulmonary resuscitation	116,920 (9.7%)	38,122 (10.0%)	**<0.001**
Recurrent myocardial infarction	4550 (0.4%)	2015 (0.5%)	**<0.001**
Pneumonia	108,140 (8.9%)	51,923 (13.7%)	**<0.001**
Deep venous thrombosis and/or thrombophlebitis	7350 (0.6%)	2489 (0.7%)	**0.001**
Pulmonary embolism	5471 (0.5%)	1893 (0.5%)	**<0.001**
Acute kidney injury	69,388 (5.7%)	38,474 (10.1%)	**<0.001**
Shock	140,523 (11.6%)	50,989 (13.4%)	**<0.001**
Stroke (ischemic or hemorrhagic)	25,693 (2.1%)	11,880 (3.1%)	**<0.001**
Intracerebral bleeding	3209 (0.3%)	1001 (0.3%)	0.915
Gastro-intestinal bleeding	13,681 (1.1%)	5466 (1.4%)	**<0.001**
Transfusion of blood constituents	115,689 (9.5%)	50,962 (13.4%)	**<0.001**

**Fig. 1:**
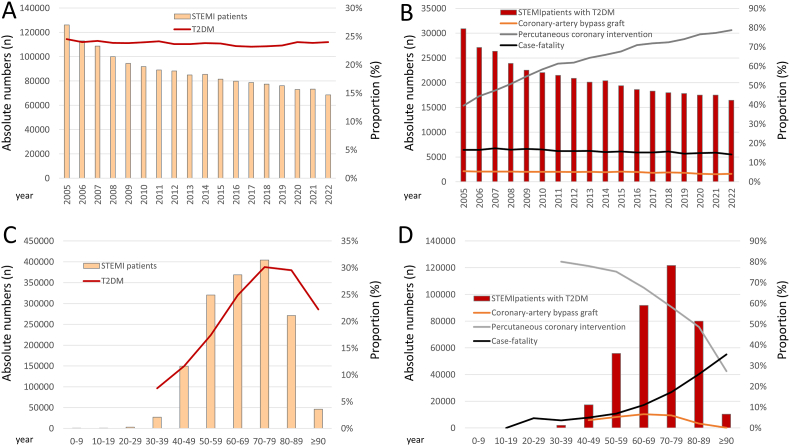
Temporal trends regarding absolute numbers of patient-cases with ST-elevation myocardial infarction (STEMI) and proportion of diabetes mellitus type 2 (T2DM) cases and regarding revascularization treatments as well as case-fatality in STEMI patients with T2DM A- Annual trends regarding absolute numbers of patient-cases with ST-elevation myocardial infarction (STEMI) and proportion of diabetes mellitus type 2 (T2DM) cases B- Annual trends regarding absolute numbers of patient-cases with STEMI in co-prevalence with T2DM and case-fatality as well as proportions of interventional and surgical revascularization treatments C- Age trends regarding absolute numbers of patient-cases with ST-elevation myocardial infarction (STEMI) and proportion of diabetes mellitus type 2 (T2DM) cases D- Age trends regarding absolute numbers of patient-cases with STEMI in co-prevalence with T2DM and case-fatality as well as proportions of interventional and surgical revascularization treatments.

While the annual total numbers of STEMI patient-cases decreased during the observational period from 2005 to 2022, the proportion of T2DM patient-cases was widely constant between 23 and 25% (Fig. 1A). As expected, the absolute numbers of STEMI patient-cases increased with age in parallel with the proportion of T2DM in the STEMI patients (Fig. 1C).

### 3.1 Comparison of STEMI patients with and without type 2 diabetes mellitus

STEMI patients with T2DM were older (71.0 [62.0-79.0] vs. 66.0 [55.0-77.0] years, P < 0.001), more often female (37.7% vs. 30.7%, P < 0.001) and revealed a distinctly higher prevalence of all investigated cardiovascular risk factors (Table 1). STEMI patients with T2DM showed a higher comorbidity burden compared to patients without T2DM with an increased prevalence of cardiovascular diseases such as heart failure (43.8% vs. 31.6%, P < 0.001), atrial fibrillation/flutter (20.7% vs. 13.8%, P < 0.001), peripheral artery disease (7.4% vs. 3.1%, P < 0.001), but also higher prevalence of comorbidities affecting other organ systems e.g. chronic obstructive pulmonary disease (7.0% vs. 5.0%, P < 0.001) and renal failure (32.1% vs. 16.4%, P < 0.001) (Table 1).

### 3.2 Impact of T2DM on reperfusion strategies

Interventional treatments comprising cardiac catheter (67.6% vs. 72.5%, P < 0.001) and PCI (61.2% vs. 66.7%, P < 0.001) were less often used in STEMI patients with T2DM compared to STEMI patients without T2DM. In contrast, coronary artery bypass grafting (CABG, 5.0% vs. 3.9%, P < 0.001) (Table 1).

Fig. 1B illustrates the sharp increase regarding usage of PCI and the usage over the years, whereas CABG slightly decreased between 2005 and 2022. Although the highest absolute numbers of STEMI patients with T2DM were seen to be admitted between the 7th and 9th decade of life, interventional revascularization treatments decreased with inclining age (Fig. 1D).

Multivariate logistic regression analyses confirmed lower usage of cardiac catheter (OR 0.89 [95%CI 0.88-0.90], P < 0.001) and PCI (OR 0.89 [95%CI 0.89-0.90], P < 0.001) in patients hospitalized due to STEMI with T2DM independently of age, sex, cardiovascular risk factors and comorbidities, whereas CABG was more often performed in STEMI patients with T2DM (OR 1.13 [95%CI 1.11-1.15), P < 0.001) (Table 2).

**Table 2:** Impact of type 2 diabetes mellitus on in-hospital adverse events of patients hospitalized due to ST-elevation myocardial infarction (STEMI) (excluding patients with T1DM) (univariate and multivariate logistic regression model).

	Univariate regression model		Multivariate regression model^a^		Multivariate regression model^b^		Multivariate regression model^c^	
	OR (95% CI)	P-value	OR (95% CI)	P-value	OR (95% CI)	P-value	OR (95% CI)	P-value
Treatment								
Cardiac catheter	0.791 (0.785-0.797)	**<0.001**	0.917 (0.910-0.925)	**<0.001**	0.887 (0.880-0.895)	**<0.001**	0.889 (0.881-0.896)	**<0.001**
Percutaneous coronary intervention	0.789 (0.783-0.795)	**<0.001**	0.910 (0.903-0.917)	**<0.001**	0.884 (0.877-0.891)	**<0.001**	0.892 (0.885-0.899)	**<0.001**
Coronary-artery bypass graft	1.298 (1.276-1.321)	**<0.001**	1.338 (1.314-1.361)	**<0.001**	1.210 (1.189-1.232)	**<0.001**	1.133 (1.113-1.154)	**<0.001**
**Adverse events during hospitalization**								
In-hospital death	1.237 (1.225-1.250)	**<0.001**	1.033 (1.022-1.044)	**<0.001**	1.200 (1.187-1.214)	**<0.001**	1.026 (1.014-1.037)	**<0.001**
Recurrent myocardial infarction	1.416 (1.344-1.493)	**<0.001**	1.393 (1.321-1.469)	**<0.001**	1.372 (1.301-1.448)	**<0.001**	1.240 (1.175-1.309)	**<0.001**
Pneumonia	1.618 (1.600-1.636)	**<0.001**	1.474 (1.458-1.491)	**<0.001**	1.532 (1.515-1.550)	**<0.001**	1.172 (1.158-1.187)	**<0.001**
Deep venous thrombosis or thrombophlebitis	1.082 (1.034-1.132)	**0.001**	1.055 (1.007-1.104)	**0.023**	1.048 (1.000-1.097)	**0.049**	0.924 (0.882-0.969)	**0.001**
Pulmonary embolism	1.105 (1.049-1.165)	**<0.001**	1.025 (0.972-1.080)	0.362	1.055 (1.000-1.113)	**0.049**	0.916 (0.868-0.967)	**0.001**
Acute kidney injury	1.858 (1.834-1.882)	**<0.001**	1.670 (1.648-1.692)	**<0.001**	1.758 (1.734-1.781)	**<0.001**	1.546 (1.525-1.568)^d^	**<0.001**
Shock	1.183 (1.171-1.196)	**<0.001**	1.112 (1.100-1.124)	**<0.001**	1.241 (1.227-1.255)	**<0.001**	0.972 (0.961-0.984)	**<0.001**
Stroke (ischemic or hemorrhagic)	1.492 (1.459-1.525)	**<0.001**	1.338 (1.309-1.368)	**<0.001**	1.369 (1.338-1.400)	**<0.001**	1.226 (1.198-1.254)	**<0.001**
Intracerebral bleeding	0.996 (0.928-1.069)	0.915	0.960 (0.894-1.031)	0.259	0.987 (0.918-1.061)	0.716	0.886 (0.823-0.953)	**0.001**
Gastro-intestinal bleeding	1.280 (1.240-1.321)	**<0.001**	1.145 (1.110-1.182)	**<0.001**	1.192 (1.154-1.231)	**<0.001**	0.971 (0.940-1.003)	0.076
Transfusion of blood constituents	1.470 (1.454-1.486)	**<0.001**	1.351 (1.336-1.366)	**<0.001**	1.362 (1.346-1.377)	**<0.001**	1.079 (1.067-1.092)	**<0.001**

a Adjusted for age and sex.

b Adjusted for age, sex, essential arterial hypertension, obesity, and hyperlipidemia.

c Adjusted for age, sex, cancer, heart failure, chronic obstructive pulmonary disease, essential arterial hypertension, acute and chronic kidney disease, atrial fibrillation/flutter, and hyperlipidemia.

d Adjusted for age, sex, cancer, heart failure, chronic obstructive pulmonary disease, essential arterial hypertension, atrial fibrillation/flutter, and hyperlipidemia.

### 3.3 Differences in adverse in-hospital events

In-hospital case-fatality of STEMI patients with T2DM was approximately 3% higher than in patients who did not suffer from T2DM (16.0% vs. 13.3%, P < 0.001). While the outcomes of recurrent myocardial infarction (0.5% vs. 0.4%, P < 0.001) and deep venous thrombosis and/or thrombophlebitis and pulmonary embolism were only slightly higher in patients with T2DM, other adverse in-hospital outcomes such as pneumonia (13.7% vs. 8.9%, P < 0.001), stroke (3.1% vs. 2.1%, P < 0.001), and bleeding events requiring transfusion of blood constituents (13.4% vs. 9.5%, P < 0.001) might be primarily accountable for higher short-term mortality in STEMI patients with T2DM (Table 1). While the case-fatality of STEMI patients with T2DM increased substantially with age, the case-fatality revealed a down-trend during the observational period 2005-2022 (Fig. 1 B and D).

In multivariate logistic regression analysis, the presence of T2DM was significantly associated with increased case-fatality (OR 1.03 [95%CI 1.01-1.04], P < 0.001) in the gender-neutral analysis after adjustment for age, sex and comorbidities (Table 2, Table 3). This association remained stable after additional adjustment for PCI (Table 3). In the sex-specific analysis, we detected an independent association of T2DM with increased case-fatality in female STEMI patients (OR 1.05 [95%CI 1.04-1.06], P < 0.001), but not in male patients (OR 0.96 [95%CI 0.95-0.97], P < 0.001) after adjustment for age, cancer, heart failure, chronic obstructive pulmonary disease, essential arterial hypertension, acute and chronic kidney disease, atrial fibrillation/flutter, and hyperlipidemia. After additional adjustment for PCI, T2DM was independently associated with increased case-fatality in females (OR 1.69 [1.64-1.74], P < 0.001), but not in males (OR 1.01 [1.00-1.03], P = 0.127) (Table 3).

**Table 3:** Impact of type 2 diabetes mellitus on in-hospital case-fatality of patients hospitalized due to ST-elevation myocardial infarction (STEMI) (excluding patients with T1DM) (univariate and multivariate logistic regression model).

	Univariate regression model		Multivariate regression model^a^		Multivariate regression model^b^		Multivariate regression model^c^	
	OR (95% CI)	P-value	OR (95% CI)	P-value	OR (95% CI)	P-value	OR (95% CI)	P-value
**Both sexes**								
In-hospital death	1.237 (1.225-1.250)	**<0.001**	1.026 (1.014-1.037)	**<0.001**	1.174 (1.162-1.186)	**<0.001**	1.015 (1.003-1.026)	**0.012**
**Sex-specific analysis**								
**Male patients**								
In-hospital death	1.201 (1.192-1.210)	**<0.001**	0.960 (0.952-0.967)	**<0.001**	1.211 (1.195-1.228)	**<0.001**	1.012 (0.997-1.027)	0.127
**Female patients**								
In-hospital death	1.215 (1.205-1.226)	**<0.001**	1.052 (1.042-1.061)	**<0.001**	1.063 (1.046-1.080)	**<0.001**	1.685 (1.635-1.737)	**<0.001**

a Adjusted for age, cancer, heart failure, chronic obstructive pulmonary disease, essential arterial hypertension, acute and chronic kidney disease, atrial fibrillation/flutter, and hyperlipidemia.

b Adjusted for percutaneous coronary intervention.

c Adjusted for age, sex, cancer, heart failure, chronic obstructive pulmonary disease, essential arterial hypertension, acute and chronic kidney disease, atrial fibrillation/flutter, hyperlipidemia and percutaneous coronary intervention.

In addition, we detected an age-dependent impact of T2DM on case-fatality in STEMI patients. The negative impact of T2DM in STEMI patients was especially detected in the 5th to 9th decade of life. In younger and older patients, T2DM seemed to have a minor influence (Table 4).

**Table 4:** Impact of type 2 diabetes mellitus on in-hospital case-fatality of patients hospitalized due to ST-elevation myocardial infarction (STEMI) in the different age-decades (excluding patients with T1DM) (univariate and multivariate logistic regression model).

Age decade	Univariate regression model		Multivariate regression model^a^	
	OR (95% CI)	P-value	OR (95% CI)	P-value
20-29 years	1.026 (0.412-2.560)	0.955	1.012 (0.368-2.784)	0.982
30-39 years	0.926 (0.728-1.178)	0.531	1.144 (0.880-1.486)	0.314
40-49 years	1.154 (1.072-1.242)	**<0.001**	1.190 (1.098-1.290)	**<0.001**
50-59 years	1.161 (1.120-1.204)	**<0.001**	1.075 (1.032-1.119)	**<0.001**
60-69 years	1.130 (1.103-1.158)	**<0.001**	1.028 (1.001-1.056)	**0.042**
70-79 years	1.079 (1.060-1.098)	**<0.001**	−1.016 (0.996-1.036)	0.109
80-89 years	0.966 (0.948-0.984)	**<0.001**	1.024 (1.004-1.045)	**0.019**
≥90 years	0.866 (0.827-0.906)	**<0.001**	0.963 (0.918-1.010)	0.122

a Adjusted for age, sex, cancer, heart failure, chronic obstructive pulmonary disease, essential arterial hypertension, acute and chronic kidney disease, atrial fibrillation/flutter, and hyperlipidemia.

Whereas the hospital setting (urban, suburban and rural) had no influence on the impact of T2DM on survival (Table S1 in the supplementary material), localization of myocardial infarction was differently affected by T2DM. While T2DM was independently associated with case-fatality in anterior wall STEMI (OR 1.03 [95%CI 1.01-1.05], P < 0.001), in posterior wall STEMI T2DM was not independently related to increased short-term mortality (OR 1.02 [95%CI 1.00-1.04], P = 0.082) (Table S2 in the supplementary material).

Regarding other adverse in-hospital outcomes, the risk for pneumonia (OR 1.17 [95%CI 1.16-1.19], P < 0.001), recurrent myocardial infarction (OR 1.24 [95%CI 1.18-1.31)], P < 0.001), stroke (OR 1.23 [95%CI 1.20-1.25], P < 0.001), and bleeding events requiring transfusion of blood constituents (OR 1.08 [95%CI 1.07-1.09)], P < 0.001) was elevated in STEMI patients with T2DM (Table 2). Remarkably, T2DM was not independently associated with increased occurrence of deep venous thrombosis and/or thrombophlebitis of the leg veins, pulmonary embolism, shock and gastrointestinal bleeding after additional adjustment for comorbidities (Table 2).

When analysing the impact of T2DM on in-hospital case-fatality, we identified that T2DM was afflicted with increased in-hospital case-fatality primarily in former years (before 2012) after adjustment for age, sex and comorbidities as well as additionally adjustment for PCI. In recent years T2DM was not independently associated with increased in-hospital case-fatality rate (Table S3 of the supplementary material).

## 4 Discussion

In the present study, more than 1.5 million hospitalizations of STEMI patients who were treated between 2005 and 2022 in Germany were included to investigate the influence of T2DM on comorbidities, in-hospital adverse events, revascularization procedures and in-hospital case-fatality. Of these cases, approximately 1/4th occurred in patients with T2DM. The findings of the present study can be summarized as follows:

Patients with T2DM presenting with STEMI have higher in-hospital mortality. The impact of T2DM on survival was more pronounced in female than in male patients and was primarily seen for anterior wall, but not in posterior wall STEMI. They also experience more complications. Part of the excess risk is related to less frequent or delayed use of interventional revascularization. STEMI patients with T2DM were older, more often female and had a higher burden of cardiovascular risk factors and comorbidities compared to patients without T2DM.

T2DM is a major risk factor for macrovascular and microvascular deviations and is a main driver of aggravated outcome in patients suffering from acute coronary syndrome and especially STEMI [2,17,44,45]. Poor clinical outcome of patients with compared to patients without diabetes mellitus was already described in patients suffering from myocardial infarction decades ago. In the early 1990's Granger et al. found a worse cardiovascular risk profile and a more severe anatomic coronary disease with higher prevalence of multivessel disease in patients suffering from myocardial infarction who additionally had diabetes mellitus [29]. In-hospital mortality of patients with diabetes mellitus was doubled compared to patients without diabetes in this study [29]. Although these results contributed significantly to the current level of knowledge, these studies were conducted more than 2 decades ago and the current status regarding the impact of T2DM on in-hospital outcomes in the light of management improvements with new treatment approaches as well as optimized standard of care of this crucial patient group of STEMI patients is not well investigated in epidemiological studies [35,46]. In accordance with the aforementioned study, our data demonstrated that T2DM was independently associated with higher case-fatality. In contrast, the impact of T2DM on in-hospital case-fatality was less intense in our study in comparison to the older study [29], which might be the beneficial result of optimized patient care of T2DM patients [35,46]. In line with our findings, an epidemiological study analyzing more than 543,000 patients with STEMI (20.8% with diabetes mellitus) of the United States National Inpatient Sample the occurrence of in-hospital sudden cardiac death was 1.7-fold increased in patients with diabetes mellitus compared to patients without diabetes mellitus [47]. In addition, an analysis of the Polish STEMI registry detected similar results to our present study with STEMI patients with diabetes mellitus being associated to older age, female sex, comorbidity and risk factor profile as well as worse coronary artery disease [48]. Mortality after more than 500 days follow-up was about doubled in STEMI patients with diabetes mellitus in this study [48]. Other studies underlines also an impact of glycometabolic state at admission on case-fatality [45,49]. Similar findings were reported in an assessment of 11 trials including 1662 patients, in which co-prevalent diabetes mellitus in patients with STEMI was related to female sex and increased age, more cardiovascular risk factors and coronary multivessel disease as well as higher mortality rates in follow-up [50]. The results of our study emphasized in accordance with the aforementioned studies that the manifestation of the impact of T2DM varied sex-specifically. T2DM afflicted the case-fatality primarily and independently of female STEMI patients, while male patients were not independently affected. In male STEMI patients the increased risk for in-hospital death was not independent of age and comorbidities. This finding supports the assumption of significant gender-gap disparities in T2DM treatment, with women often facing delayed diagnosis accompanied by higher cardiovascular risks, and less often guideline-compliment treatments and lower frequency regarding achieving treatment targets [[51], [52], [53]]. In addition, therapeutic nihilism toward older and diabetic patients among operators has to be discussed. Fig. 1D illustrates a substantial decrease regarding the use of revascularization treatments in STEMI patients with T2DM aged 70 years and older. This finding is in accordance with literature [2,54].

While the impact of T2DM on survival was not relevantly influences by the hospital type (urban vs. suburban vs. rural hospitals) and therefore management of STEMI patients with T2DM seems to be widely standardized and might follow the current guidelines [35,46], we detected a difference between myocardial infarction localization. While anterior wall myocardial infarction seems to be more prone to be affected by T2DM, posterior wall myocardial infarction was less often afflicted by T2DM implications and complications. Studies have shown on the one hand that the intense of hyperglycemia was associated with myocardial infarction size [55]. On the other hand, smaller studies reported that prediabetes and diabetes mellitus were related to smaller coronary artery diameters with diffuse coronary narrowing due to atherosclerotic deviations focusing more often on the left coronary arteries than on the right coronary artery [56]. This might be one possible explanation for the difference between the impact of T2DM on case-fatality in anterior and posterior wall myocardial infarction.

Remarkably, our study results emphasized that the negative effect of T2DM on the short-term survival of STEMI patients was primarily seen in the former years before 2012. In recent years, T2DM was in the annual analyses no more afflicted by increased in-hospital case-fatality rate independently of age, sex and comorbidities as well as PCI. This finding supports the assumption that guideline-complient therapies and the broader and earlier use of revascularization therapies improved the outcomes of STEMI patients with T2DM.

In this context, although an invasive strategy improved outcome for both diabetic and nondiabetic patients in previous studies [57], we identified an underuse of PCI treatment in STEMI patients with T2DM in comparison to those without T2DM. PCI revascularization was more than 5% less often used in STEMI patients with T2DM than without. This finding is in accordance with literature [2,54,58]. In the Atherosclerosis Risk in Communities Study Community Surveillance the underuse of revascularization therapies was even larger as in our study. This underuse of revascularization strategies (detected in our study) was primarily seen during the first years of the observational period. Fortunately, the PCI rate increased from 39.5% in the years 2005 to 78.7% in the years 2022, indicating for a more and more guideline-based optimization of STEMI patients with T2DM. It has to be underlined that it is of outstanding importance to reach a high revascularization rate in STEMI patients to improve survival of STEMI patients and especially in STEMI patients with T2DM. The reasons for lower revascularization rates are multi-factorial, but not fully illucidated. Firstly, patients with diabetes mellitus report less often chest pain at presentation [54,59,60]. This may partly explain the underuse and the delay in the use of invasive angiography and revascularization in patients with myocardial infarction and diabetes melllitus compared with patients without diabetes mellitus [54,59,60]. Secondly, patients with myocardial infartion suffer more often from complex coronary anatomy, multi-vessel involvement, and smaller vessel diameter [54,59]. In line with the literature [54], we detected an increase regarding usage of PCI and a decrease of the use of CABG between 2005 and 2022. Nevertheless, muliti-vessel disease in T2DM might hamper further extension of revascularization strategies. Thirdly, it has been reported that incidence of type II myocardial infarction might be higher in patients with diabetes mellitus [54]. Since type II myocardial infarction is caused by a supply-demand mismatch of oxygen to the heart without acute plaque rupture accounting for 10–20% of the cases [54], therefore, the underuse of revascularization may in part be reflective of type II myocardial infarction cases. In parallel with previous studies, our study results revealed a higher burden of cardiovascular risk factors and comorbidities in STEMI patients with T2DM [29,45,48,50]. As expected, T2DM was associated with higher prevalence of cardiovascular diseases, renal and pulmonary diseases [[1], [2], [3],^44^,^61^]. Regarding adverse in-hospital events, the results of our present study revealed that presence of T2DM was associated with higher rates of stroke, pneumonia, and acute renal failure. In contrast, T2DM was not independently associated with increased occurrence of deep venous thrombosis and/or thrombophlebitis of the leg veins, pulmonary embolism, shock and gastrointestinal bleeding after additional adjustment for comorbidities and therefore might the occurrence of these adverse in-hospital outcomes related to one other important comorbid condition. In this context, T2DM was independently associated slightly higher occurrence rate of recurrent myocardial infarction, which is in accordance with literature [31,32]. The study by Piccolo et al. investigated the impact of diabetes mellitus on mortality regarding time and type of myocardial infarction and found relevant differences [31]: Mortality 1 year after the event was highest in patients with diabetes who suffered from STEMI (13.4%) and non-ST-elevation myocardial infarction (10.3%), whereas mortality of patients without diabetes mellitus with STEMI (6.4%) and non-ST-elevation myocardial infarction (4.4%) was significantly lower. Also, diabetes mellitus was associated with early (day 0 to 30) death after STEMI in comparison to late (day 31 to 365) mortality and non-ST-elevation myocardial infarction, and co-prevalence of diabetes mellitus in STEMI was related to an increased risk of early stent thrombosis as well as target lesion revascularization within early and late follow-up. In line, a study from Denmark revealed significantly higher rates of recurrent myocardial infarction (more than doubled), target lesion revascularization (1.5-fold higher) and mortality (about doubled) in a three years follow-up in patients with compared to patients without diabetes mellitus who initially were treated with PCI due to STEMI, whereas no difference was found regarding stent-thrombosis between both groups [32]. A study from the United States revealed similar findings as the present study with increased in-hospital mortality in patients with diabetes mellitus as well as worse comorbidity profile and lower revascularization performance, and furthermore showed higher mortality rates even after 1 year and 5 years after STEMI [18]. An analysis from an Italian database demonstrated similar findings with STEMI patients with diabetes mellitus being more likely female, bearing more comorbidities and higher rates of death, re-infarction and stent thrombosis after an investigation period of 1200 ± 440 days [62]. The largest follow-up time-frame on the impact of diabetes mellitus on clinical outcome in patients with STEMI was recently published by Spione et al. [17]: In their 10-years follow-up the authors found higher incidences of any revascularization (not target vessel revascularization) in the diabetes group, which mainly occurred within the first 5 years after the event. No differences between patients with and without diabetes mellitus 10 years after STEMI were found concerning all-cause and cardiac mortality, any myocardial infarction as well as target lesion and target vessel revascularization.

In line with previously published studies [63,64], the adverese in-hospital events shock and bleeding events occurred more often in STEMI patients with T2DM. While the univariate logistic regressions were able to confirm this association between T2DM and shock as well as bleeding events [[63], [64], [65], [66], [67]], the multivariate regression analyses failed to demonstrated independent assocaitions of T2DM with increased occurrence of shock as well as intracranial and gastrointestinal bleeding events. This finding might be mainly driven by the adjustment of the regression model in the light of higher age and comorbidity burden in STEMI patients with T2DM.

In addition, also venous thromboembolism events were more often recognized in STEMI patients with T2DM. Since the incidence of venous thromboembolism increases significantly with age [39,[68], [69], [70], [71]], it is no surprise that - after adjusting the logistic regressions for age and age-related comorbidities - the multivariate regressions do not show an independent asociation between T2DM and increased rates of venous thromboembolism in STEMI patients, although it is well known that myocardial infarction is a risk factor for the occurrence of venous thromboembolic events [72,73].

Summarizing the literature and taking into account our study results, T2DM decreases the survival of STEMI patients during short and long-term, whereby the impact of T2DM might be especially high within the first five years after the acute event compared to patients without diabetes mellitus. Nevertheless, our data suggest that the rise in the use of revascularization therapies and especially in PCI are accompanied by improved outcomes of STEMI patients with T2DM. This has to be recognized as a favourable effect of guideline-complient treatment. In this context, a guideline-based therapy with timely revascularization of the primary or recurrent myocardial infarction is of outstanding interest to optimize myocardial perfusion, avoid early death and prevent complications.

Our findings highlight the particular vulnerability of STEMI patients with T2DM, underlining the need for intensified monitoring and guideline-based secondary prevention. Therefore, in this group special attention by medical personnel and adjusted strategies are required like the use of drug eluting stents rather than bare metal stents [37,62,74], optimal glucose management without hypoglycemia, since this is associated with increased mortality in the setting of STEMI in patients with and without diabetes mellitus [75], optimization of comorbidities and best possible secondary prevention including continuous medical monitoring. Also, further effort is required to identify reasons for and find solutions against the worse initial outcome of STEMI patients with diabetes mellitus aiming to improve outcome of this crucial vulnerable patient-group.

## 5 Limitations

There are some limitations that merit consideration: First, the study results are based on ICD as well as OPS discharge codes of hospitalized patients, which might lead to an under-reporting/under-coding. The under-reporting and under-coding have to be mentioned as key limitations of these epidemiological studies with administrative data-sets. Although, it has to be expected that expensive diagnosis and procedures will not be forgotten in coding since these will trigger higher remuneration, it has to be suggested that the coding of diagnosis and procedures that are not related to higher reimbursement might be less strictly coded. Second, we can only provide data from the timeframe of hospitalization and have no data about later follow-ups. Third, data regarding disease duration of T2DM are not available in the German nationwide inpatient statistics. Fourth, as an observational study which is based on reporting of hospitals by DRG coding, the German nationwide inpatient sample cannot deliver information about underlying mechanistic insights/factors of T2DM leading to increased morbidity or mortality. Fifth, the dataset of the German nationwide inpatient statistics has no access to laboratory results such as cardiac Troponins or NT-pro-BNP levels. Thus, we were not able to provide correlations between the levels of these laboratory makers and survival. Sixth, the medications of the patients are not available in the dataset of the German nationwide inpatient statistics. Seventh, the causes of death are not available in the dataset of the German nationwide inpatient statistics.

## 6 Conclusions

Patients with T2DM suffering from STEMI are associated with an aggravated comorbidity-profile, increased risk for in-hospital adverse events, lower performance of revascularization, and increased in-hospital mortality compared to individuals without T2DM. This vulnerable patient group therefore needs special attention during and after hospital-stay with continuous medical monitoring and post-myocardial infarction secondary prevention strategies.

## CRediT authorship contribution statement

**Volker H. Schmitt:** Writing – review & editing, Writing – original draft, Conceptualization. **Omar Hahad:** Writing – review & editing. **Visvakanth Sivanathan:** Writing – review & editing. **Christoph Brochhausen:** Writing – review & editing. **Christine Espinola-Klein:** Writing – review & editing. **Thomas Münzel:** Writing – review & editing. **Philipp Lurz:** Writing – review & editing. **Tommaso Gori:** Writing – review & editing. **Lukas Hobohm:** Writing – review & editing, Validation, Supervision, Software, Project administration, Methodology, Investigation, Formal analysis, Conceptualization. **Karsten Keller:** Writing – review & editing, Writing – original draft, Visualization, Validation, Supervision, Software, Resources, Project administration, Methodology, Investigation, Formal analysis, Data curation, Conceptualization.

## Funding

None.

## Conflicts of interest

VHS, OH, VS, CB, TM and KK have no conflicts of interest. CEK reports having received honoraries from Amarin Germany, Amgen GmbH, Bayer Vital, Boehringer Ingelheim, Bristol-Myers Squibb, Daiichi Sankyo, Leo Pharma, MSD Sharp & Dohme, Novartis Pharma, Pfizer Pharma GmbH, Sanofi-Aventis GmbH, outside the submitted work. PL has received institutional fees and research grants from Abbott Vascular, Edwards Lifesciences, and ReCor, honoraria from Edwards Lifesciences, Abbott Medical, Innoventric, ReCor and Boehringer Ingelheim and has stock options with Innoventric. TG has received grant support from Abbott Vascular and Shockwave and speaker's honoraria from Novartis, Shockwave, Abbott Vascular, Astra Zeneca, BMS, MSD, Bayer outside this work. LH received lecture/consultant fees from MSD and Actelion, outside the submitted work. OH is a Young Scientist and TM a P.I. of the DZHK (German Center for Cardiovascular Research), Partner Site Rhine-Main, Mainz, Germany. OH and TM are supported by the environmental network EXPOHEALTH funded by the Science Initiative of the state Rhineland-Palatinate, Germany, and by the environmental research consortium MARKOPOLO, which is funded by the European Union (Grant Agreement Number 101156161) and the Swiss State Secretariat for Education, Research and Innovation (SERI). Views and opinions expressed are, however, those of the authors only and do not necessarily reflect those of the European Union, the European Health and Digital Executive Agency (HADEA) or the SERI. Neither the European Union nor the granting authorities can be held responsible for them. The funders had no role in the paper's design, interpretation, and writing.
