# Supplementary material for: Impact of type 2 diabetes mellitus on in-hospital outcomes in patients with ST-elevation myocardial infarction: A nationwide study in Germany
Source: Metabol Open. 2026 Mar 25;30:100462. doi: 10.1016/j.metop.2026.100462 (PMC13054436; doi:10.1016/j.metop.2026.100462)
Supplement: Multimedia component 1 [file mmc1.pdf]

Supplementary material

Impact of Type 2 Diabetes Mellitus on In-Hospital Outcomes in Patients with ST-Elevation Myocardial Infarction: A Nationwide Study in Germany

Volker H. Schmitt, MD[1]; Omar Hahad, PhD[1,2]; Visvakanth Sivanathan, MD[3]; Christoph Brochhausen, MD[4]; Christine Espinola-Klein, MD[1,3]; Thomas Münzel, MD[1,2]; Philipp Lurz, MD[1,2]; Tommaso Gori, MD[1,2,3]; Lukas Hobohm, MD[1,3]; Karsten Keller, MD[1,3]

[1] Department of Cardiology, University Medical Center of the Johannes Gutenberg-University of Mainz, Germany

[2] German Center for Cardiovascular Research (DZHK), Partner Site Rhine Main, Mainz, Germany

[3] Center for Thrombosis and Hemostasis (CTH), University Medical Center of the Johannes Gutenberg-University of Mainz, Germany

[3] Department of Gastroenterology, University Medical Center of the Johannes Gutenberg-University of Mainz, Germany

[4] Institute of Pathology, Medical Faculty Mannheim, Heidelberg University, Germany

Table S1: **Impact of type 2 diabetes mellitus on in-hospital case-fatality of patients hospitalized due to ST-elevation myocardial infarction (STEMI) stratified by hospital type (urban vs. suburban vs. rural hospital) (excluding patients with T1DM)**

	**Univariate regression model**	
	**OR (95% CI)**	**P-value**
**Urban hopitals**	1.152 (1.140-1.163)	**<0.001**
**Suburban hospitals**	1.218 (1.203-1.233)	**<0.001**
**Rural hospitals**	1.246 (1.228-1.264)	**<0.001**

Table S2: **Impact of type 2 diabetes mellitus on in-hospital case-fatality of patients hospitalized due to ST-elevation myocardial infarction (STEMI) in the different STEMI subtype (excluding patients with T1DM) (univariate and multivariate logistic regression model)**

**Age decade**	**Univariate regression model**		**Multivariate regression model ***	
	**OR (95% CI)**	**P-value**	**OR (95% CI)**	**P-value**
Anterior wall STEMI	1.212 (1.194-1.230)	**<0.001**	1.031 (1.014-1.048)	**<0.001**
Posterior wall STEMI	1.293 (1.271-1.316)	**<0.001**	1.017 (0.998-1.037)	0.082
STEMI of other localisation	1.198 (1.161-1.236)	**<0.001**	1.011 (0.976-1.048)	0.535
STEMI of not classified localisation	0.943 (0.908-0.979)	**0.002**	0.936 (0.898-0.976)	**0.002**

* Adjusted for age, sex, cancer, heart failure, chronic obstructive pulmonary disease, essential arterial hypertension, acute and chronic kidney disease, atrial fibrillation/flutter, and hyperlipidemia

Table S3: **Impact of type 2 diabetes mellitus on in-hospital case-fatality of patients hospitalized due to ST-elevation myocardial infarction (STEMI) in the different treatment years 2005-2022 (excluding patients with T1DM) (univariate and multivariate logistic regression model)**

Year	Univariate regression model		Multivariate regression model *		Multivariate regression model **	
	**OR (95% CI)**	**P-value**	**OR (95% CI)**	**P-value**	**OR (95% CI)**	**P-value**
2005	1.289 (1.244-1.335)	**<0.001**	1.067 (1.026-1.109)	**0.001**	1.040 (0.999-1.082)	0.054
2006	1.268 (1.222-1.317)	**<0.001**	1.053 (1.010-1.098)	**0.015**	1.032 (0.989-1.077)	0.143
2007	1.310 (1.262-1.360)	**<0.001**	1.089 (1.044-1.135)	**<0.001**	1.066 (1.022-1.112)	**0.003**
2008	1.235 (1.187-1.285)	**<0.001**	1.030 (0.986-1.077)	0.186	1.007 (0.963-1.053)	0.755
2009	1.267 (1.216-1.319)	**<0.001**	1.047 (1.000-1.096)	**0.048**	1.024 (0.978-1.072)	0.311
2010	1.331 (1.277-1.388)	**<0.001**	1.094 (1.044-1.146)	**<0.001**	1.081 (1.032-1.133)	**0.001**
2011	1.298 (1.244-1.355)	**<0.001**	1.055 (1.006-1.107)	**0.028**	1.055 (1.005-1.107)	**0.031**
2012	1.274 (1.219-1.330)	**<0.001**	1.038 (0.989-1.090)	0.131	1.030 (0.981-1.082)	0.231
2013	1.261 (1.207-1.318)	**<0.001**	1.046 (0.996-1.098)	0.075	1.040 (0.990-1.093)	0.122
2014	1.248 (1.194-1.304)	**<0.001**	1.040 (0.989-1.092)	0.124	1.036 (0.986-1.089)	0.158
2015	1.273 (1.216-1.332)	**<0.001**	1.034 (0.983-1.087)	0.198	1.030 (0.979-1.083)	0.261
2016	1.223 (1.167-1.281)	**<0.001**	1.021 (0.969-1.075)	0.434	1.021 (0.969-1.076)	0.434
2017	1.182 (1.128-1.239)	**<0.001**	0.970 (0.921-1.023)	0.262	0.971 (0.921-1.024)	0.277
2018	1.233 (1.177-1.292)	**<0.001**	1.037 (0.984-1.093)	0.173	1.032 (0.979-1.087)	0.243
2019	1.143 (1.089-1.199)	**<0.001**	0.962 (0.911-1.014)	0.151	0.956 (0.906-1.009)	0.104
2020	1.142 (1.089-1.199)	**<0.001**	0.972 (0.921-1.026)	0.303	0.974 (0.923-1.028)	0.345
2021	1.109 (1.057-1.164)	**<0.001**	0.970 (0.919-1.023)	0.261	0.969 (0.918-1.023)	0.255
2022	1.039 (0.987-1.092)	0.142	0.902 (0.852-0.955)	**<0.001**	0.901 (0.851-0.954)	**<0.001**

* Adjusted for age, sex, cancer, heart failure, chronic obstructive pulmonary disease, essential arterial hypertension, acute and chronic kidney disease, atrial fibrillation/flutter, and hyperlipidemia

** Adjusted for age, sex, cancer, heart failure, chronic obstructive pulmonary disease, essential arterial hypertension, acute and chronic kidney disease, atrial fibrillation/flutter, hyperlipidemia and percutaneous coronary intervention
